# Efficacy and safety of cadonilimab (PD-1/CTLA-4 bi-specific antibody) and adjuvant anti-angiogenesis therapy in treated, recurrent, or metastatic cervical cancer

**DOI:** 10.3389/fonc.2025.1614434

**Published:** 2025-06-30

**Authors:** Fang Zhou, Shushu Liu, Ying Zuo, Danial F. Naseem, Yinguang Li, Yin Wang, Cuicui Meng, Yipeng Song

**Affiliations:** ^1^ Department of Radiotherapy, The Affiliated Yantai Yuhuangding Hospital of Qingdao University, Yantai, China; ^2^ Department of Gynecology, The Affiliated Yantai Yuhuangding Hospital of Qingdao University, Yantai, China; ^3^ Department of Head and Neck Surgery, MD Anderson Cancer Center, Houston, TX, United States; ^4^ Department of General Surgery, Anqiu Maternal and Child Health Hospital, Weifang, China; ^5^ Department of Otorhinolaryngology, Qilu Hospital of Shandong University, Jinan, Shandong, China; ^6^ National Health Commission (NHC) Key Laboratory of Otorhinolaryngology, Shandong University, Jinan, Shandong, China; ^7^ Department of Radiotherapy, Yantaishan Hospital, Yantai, China

**Keywords:** cervical cancer, immunotherapy, anti-angiogenesis therapy, PD-1, CTLA-4

## Abstract

**Background:**

Combined immunotherapy and antiangiogenic therapy have exhibited synergistic antitumor effects in several cancers. The prognosis of recurrent or metastatic cervical cancer (r/mCC) is poor, especially for patients with prior multi-line treatments. This study aimed to evaluate the efficacy and safety of cadonilimab(PD-1/CTLA-4 bispecific)with anti-angiogenesis adjuvant therapy (bevacizumab or anlotinib) in pretreated patients with r/mCC.

**Methods:**

Nineteen patients treated with cadonilimab plus bevacizumab or anlotinib with or without chemotherapy were included. Cadonilimab was administered at dose of 10mg/kg intravenously. Patients receiving anti-angiogenic therapy received either bevacizumab or anlotinib administered orally. The safety, objective response rate (ORR), progression-free survival (PFS) and overall survival (OS) were assessed.

**Results:**

The median follow-up was 15.5 months study patients. Among all 19 patients, 2 patients (10.5%) achieved complete response (CR), 2 patients (10.5%) achieved partial response (PR) and 4 patients (21.1%) achieved stable disease (SD), with an ORR of 21.1% and DCR of 42.1%. Moreover, the median PFS was 10.5 months (95% CI: 6.1-14.9 months) and 1-year OS rate was 78.3%. Proteinuria (47.4%) and hypertension (42.2%) were the most common treatment-related adverse events (TRAE), with 5 (26.3%) patients experiencing Grade 3 TRAEs, while no treatment related deaths were observed.

**Conclusions:**

This is the first report exploring the efficacy and safety of treating patients concurrently with Cadonilimab plus bevacizumab or anlotinib with heavily pretreated r/mCC. The findings suggest that this regimen might be potentially efficacious and safe with relatively manageable toxicity. Further trials with a control arm are required to validate our findings.

## Introduction

Despite the availability of adequate screening protocols and prevention techniques, such as HPV vaccination, cervical cancer is still the fourth most common cancer affecting women worldwide (1, 2). Early stage and localized cancers maybe cured by surgery, chemoradiotherapy, or both, but recurrent, metastatic, or persistent cases are often incurable (3–5). For these patients, platinum-based chemotherapy with bevacizumab, if not contraindicated, is standard of care (6). Unfortunately, this regimen only provides a median overall survival (OS) of 14.3–18.3 months (7, 8), and, until recently, there was little benefit seen with second-line systemic therapies.

Immunotherapy, represented by PD-1 inhibitors for cervical cancer, has shown promising efficacy in multiple studies (9, 10). The pivotal KEYNOTE-028 trial, first established pembrolizumab as second-line treatment for patients with PD-L1-positive persistent or r/mCC. The KEYNOTE-158 trial demonstrated the efficacy of pembrolizumab monotherapy with an ORR of 14.3% (11, 12). These trials established the feasibility of targeting the PD-1 and PD-L1 immune checkpoint axis as a therapeutic approach in r/mCC. Still, only a small percentage of patients responded to single-agent immunotherapy (13). Therefore, combination immunotherapy with various treatments such as chemotherapy and/or anti-angiogenesis is now widely utilized.

Cadonilimab, a PD-1/CTLA-4 bi-specific blocker being developed for the treatment of a range of solid tumors, was the first dual immune checkpoint inhibitor treatment to be approved (14). Cadonilimab showed good antitumor efficacy in a phase Ib/II study in patients with r/mCC who had failed previous platinum-containing chemotherapy (15, 16). Gynecologic Oncology Group 240 trial has shown that incorporation of bevacizumab to chemotherapy regimens improved OS in the first-line setting (6). Anlotinib, a multikinase inhibitor with broad inhibitory effects on oncoangiogenesis and tumor growth, has demonstrated its efficacy and safety in Chinese patients with r/mCC in a phase II clinical study (17). At present, although preclinical trials have shown that the combined immunotherapeutic agents and antiangiogenic inhibitors targeting VEGF, FEGF, EGFR and other signaling pathways have exhibited synergistic antitumor effects in several cancer types (18), the safety and efficacy of cadonilimab with adjuvant bevacizumab or anlotinib are still unknown.

Because of these considerations, we first investigated the efficacy and safety of cadonilimab with either bevacizumab or anlotinib as second-line or later therapy in r/mCC.

## Methods

### Study patients

In this retrospective study, the patients with r/mCC (age at 18–75 years old) without considering PD-L1 expression status and with prior failure of at least one line of systemic therapy (including anti-PD-1 antibodies) or could not tolerate chemotherapy were eligible. The patients must have at least one measurable lesion per RECIST v1.1, adequate organ function, and an Eastern Cooperative Oncology Group performance status score of no more than 2. The patients with significantly autoimmune disease were excluded. The patients were also ineligible if they had non-healing wounds or active bleeding conditions. All patients in our study received treatment with cadonilimab and either bevacizumab or anlotinib. These patients were included irrespective of any concurrent treatment with chemotherapy. All patients started treatment at Yantai Yuhuangding Hospital and Yantaishan Hospital between July 2022 and January 2024. Pertinent patient data, including baseline characteristics, tumor history, prior therapies, adverse events, treatment response, and outcomes were analyzed.

### Patient treatment

Patients were administered cadonilimab 10mg/kg intravenously once on day 1 plus bevacizumab 7.5 to 10 mg/kg intravenously on day 1 or anlotinib 12 mg orally once daily on days 1–14 every 3 weeks. Treatment of cadonilimab was continued until disease progression was noted or unacceptable toxic effects were observed. No dose adjustment was performed for patients receiving cadonilimab. We modified the bevacizumab dose only when weight of the patient changed by more than 10%. Bevacizumab treatment was delayed or discontinued for patients depending on the severity of uncontrolled hypertension, severe bleeding, proteinuria (urine protein to creatinine ratio of ≥3.5), arterial or venous thrombosis, coagulopathy, and intestinal obstruction or disruption. The dose of anlotinib was reduced in patients with grade 3 or 4 treatment-related toxicities. If the patients were still intolerant, the dose was adjusted from 10mg to 8mg. The decision to treat patients adjunctly with chemotherapy was made in accordance to each patient’s condition. By closely monitoring the patients’ adverse events and adjusting the dose of bevacizumab and anlotinib accordingly, we may be able to minimize side effects without sacrificing too much efficacy. Secondly, pre-treatment and concurrent supportive care and personalized treatment schedules to the patient’s specific needs may also be beneficial.

### Treatment response

Responses, including complete response (CR), partial response (PR), stable disease (SD) and progressive disease (PD) were assessed by investigators according to the Response Evaluation Criteria in Solid Tumors (RECIST) version 1.1 using computed tomography (CT) or magnetic resonance imaging (MRI) every 6 weeks on-study.

In patients showing disease control or sustained relief from cadonilimab, treatment was continued even if there was preliminary evidence of PD on imaging.

### Statistical analysis

The primary end point was ORR. Secondary endpoint included adverse events, PFS and OS. PFS was calculated from the initiation of cadonilimab to PD or death from any cause. OS was calculated from the initiation of cadonilimab to death from any cause. Adverse events were graded using the Common Terminology Criteria for Adverse Events (CTCAE - version 5.0). Calculations were performed using SPSS (version 24.0) statistical software system.

## Results

### Characteristics of study patients

From July 2022 to January 2024, a total of 19 patients with the diagnoses of recurrent or metastatic cervical carcinoma who had failed at least first-line therapy were treated with cadonilimab. The clinicopathologic and treatment characteristics of the 19 patients included in this study were presented in **Table 1**. Patients’ ages ranged from 42 to 74 years (median, 58.5 years). Squamous cell carcinoma was the most common histology seen in 14 patients (73.7%) followed by adeno subtype in 4 (21.1%) and adenosquamous subtype in 1 patient (5.2%), respectively. Six patients (31.5%) had PD-L1–positive tumors and 4 (21.1%) were determined to be PD-L1–negative. The remaining 9 patients (47.4%) of unknown status were not evaluable or had tissue samples that were not available. Two patient (10.5%) had recurrent disease, 13 patients (68.4%) had metastatic disease, and 4 patients (21.1%) had both recurrent and metastatic disease. The recurrence included pelvic and/or regional lymph nodes recurrence, with pelvic recurrence accounting for 50%, pelvic lymph nodes recurrence accounting for 16.7%, and retroperitoneal lymph nodes recurrence accounting for 66.7%, respectively. In total, 2 patients had only pelvic recurrence, 1 patient had both pelvic recurrence and retroperitoneal lymph nodes recurrence, 2 patients had retroperitoneal lymph nodes recurrence, and 1 patient had both pelvic lymph nodes and retroperitoneal lymph nodes recurrences. Lung and non-regional lymph nodes were the most common sites of distant metastasis (both accounting for 58.3%), followed by bone (25.0%). During the primary treatment, 16 patients (84.2%) initially underwent definitive radio-chemotherapy, and the other three had surgery followed by radiotherapy. All patients received taxane-containing first line chemotherapy. In addition, 15 patients (78.9%) had received bevacizumab and 12 (63.2%) had received PD-1 inhibitors as part of a previous therapeutic regimen. Among the 12 patients who had received prior immunotherapy before cadonilimab treatment, the interval did not vary significantly. The shortest interval was 3 weeks, and the longest was 5.2 weeks. The median interval was 3.4 weeks. A shorter interval mainly attributed to patient’s more aggressive disease course and a less effective prior immunotherapy. It is worth noting that nearly half of these patients (47.4%) had previous been treated with anti-PD-1 immunotherapy combined with anti-angiogenesis therapy, with 6 cases using anti-PD-1 immunotherapy combined with bevacizumab and 3 cases using anti-PD-1 immunotherapy combined with anlotinib. The median time from last treatment to progression was 9.9 (range 1.7-23.5) months.

**Table 1:** The characteristics of study patients at baseline.

Characteristic	N=19	%
Age (years)		
Median	58.5 (42–74)	
Histology		
Squamous	14	73.7
Adeno	4	21.1
Adenosquamous	1	5.2
ECOG PS		
0-1	8	42.1
2	11	57.9
FIGO stage at initial diagnosis		
IB1	1	5.2
IIB	4	21.1
IIIC	9	47.4
IV	5	26.3
Disease presentation		
Recurrent	2	10.5
Metastatic	13	68.4
Both Recurrent and Metastatic	4	21.1
PD-L1 expression status		
Positive (CPS ≥1%)	6	31.5
Negative (CPS <1%)	4	21.1
Unknown	9	47.4
Time from last treatment to progression, month	9.9 (1.7-23.5)	
Prior local treatment		
Radiotherapy only	16	84.2
Surgery and Radiotherapy	3	15.8
Prior lines of therapy		
1	8	42.1
2	9	47.4
3	2	10.5
Prior antineoplastic agents		
Taxane-Platinum	19	100.0
Bevacizumab	15	78.9
PD-1 inhibitors	12	63.2
Treatment pattern		
Cadonilimab + Anlotinib ± Chemo	8	42.1
Cadonilimab + Bevacizumab ± Chemo	11	57.9
No. Cadonilimab cycles	12 (4–28)	

ECOG PS, Eastern Cooperative Oncology Group performance status; FIGO, International Federation of Gynecology and Obstetrics; PD-L1, programmed death-ligand 1; CPS, Combined Positive Score.

Bevacizumab was given with a dose of 7.5 to 10 mg/kg intravenously according to the assessment of the patient’s economic factors and risk of fistula. Among the 19 patients, 8 patients (42.1%) received cadonilimab plus anlotinib, whereas 11 patients (57.9%) received cadonilimab plus bevacizumab. A total of 243 cycles of cadonilimab were completed with the mean number of cycles being 12 (range 4–28).

### Efficacy and survival

**Figure 1:**
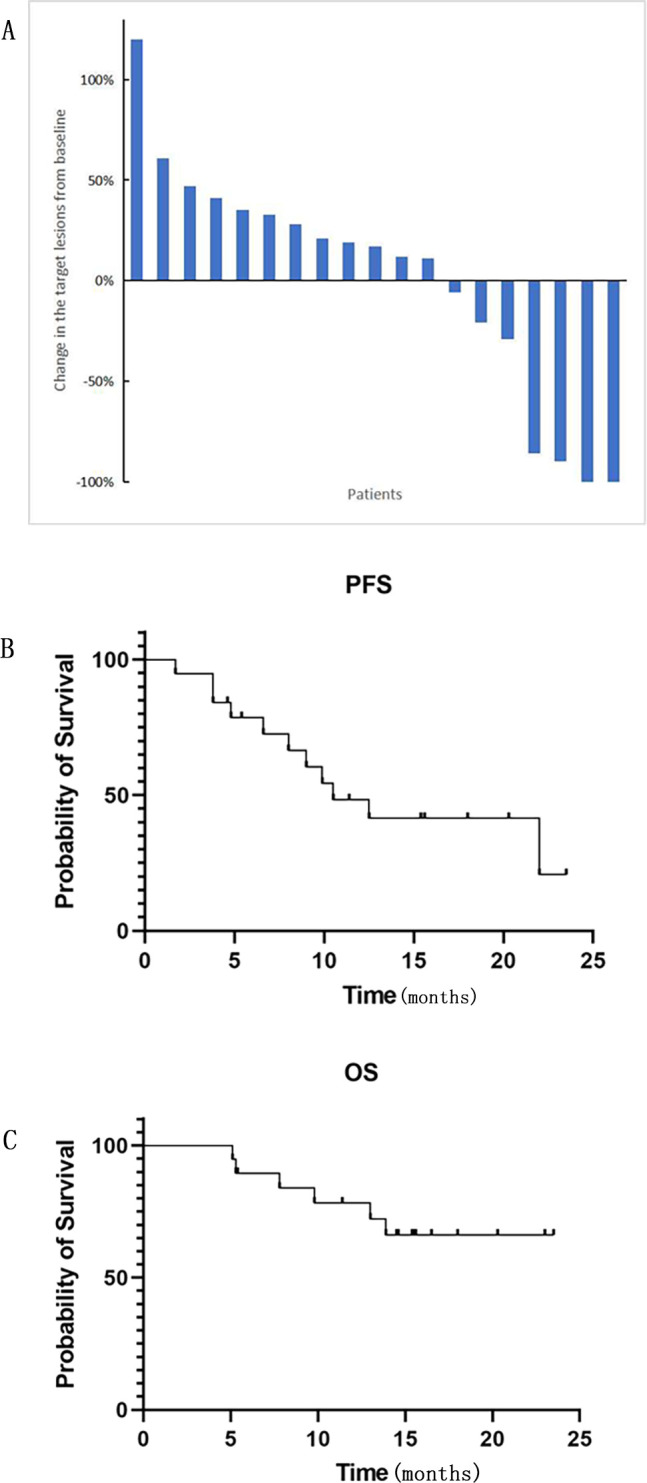
**(A)** The changes in the target lesions from baseline in tumor size (n = 19). **(B)** PFS (progression-free survival) and **(C)** OS (overall survival) were Kaplan-Meier estimates of survival outcomes. Median PFS was 10.5 months (95% CI: 6.1-14.9 months). The 6-month and 1-year OS rates were 89.5% and 78.3%, respectively.

**Figure 2:**
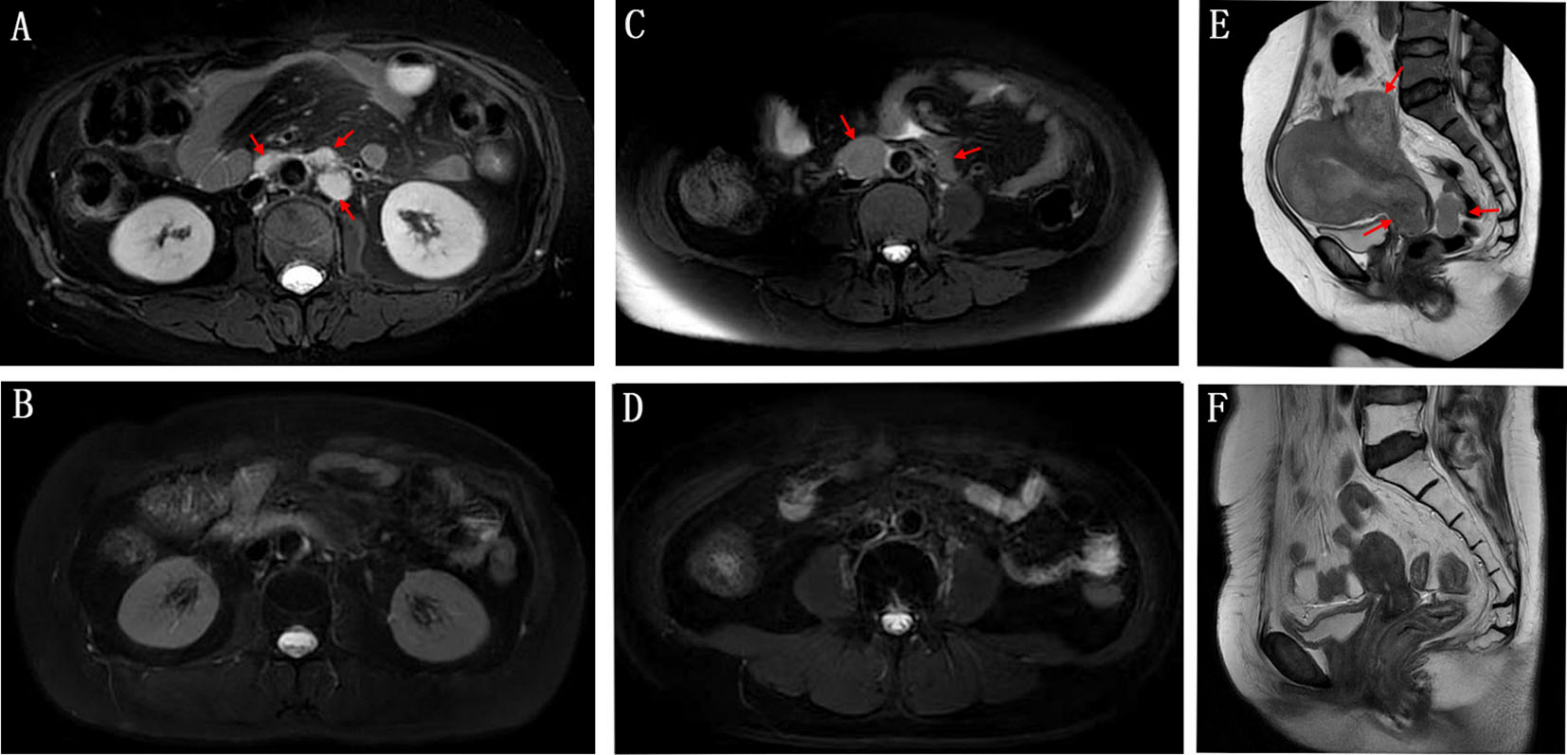
Magnetic resonance imaging (MRI) findings of a patient initially dosed in March 2023 achieved CR (complete remission) in February 2024 after 15 cycles of treatment. The patient’s condition remained stable until the end of the last follow-up. The upper panel shows the patient’s retroperitoneal metastatic lymph nodes **(A)**, pelvic metastatic lymph nodes **(C)**, and pelvic lesions **(E)** before treatment. **(B, D, F)** shows the changes after treatment.

At the end of follow-up time on July 31, 2024, 2 patients (10.5%) experienced CR, 2 patients (10.5%) achieved PR, 4 patients (21.1%) had SD, and 11 patients (57.9%) experienced PD. The ORR and DCR were 21.1% and 42.1%, respectively (**Table 2**). With respect to the 6 PD-L1 positive patients, 1 achieved PR, 2 had SD and 3 experienced PD. Among the 4 PD-L1 negative patients, 1 achieved CR and the other 3 had PD. **Figure 1A** demonstrates changes in tumor size from baseline. As of the last follow-up, 6 patients died. The median PFS was 10.5 months (95% CI: 6.1-14.9 months) (**Figure 1B**). The 6-month and 1-year OS rates were 89.5% and 78.3%, respectively (**Figure 1C**). The median overall survival time has not been reached. **Figure 2** shows the magnetic resonance imaging (MRI) findings of a patient who achieved CR.

**Table 2:** Overall response to cadonilimab based treatment (RECIST).

Overall responses	N=19	%
Complete response (CR)	2	10.5
Partial response (PR)	2	10.5
Stable disease (SD)	4	21.1
Progressive disease (PD)	11	57.9
ORR (CR+PR)	4	21.1
DCR (CR+PR+SD)	8	42.1

ORR, objective response rate; DCR, disease control rate.

### Safety

Thirteen (81.3%) patients experienced at least one treatment-related adverse event (TRAE). The most common TRAEs were proteinuria (47.4%), hypertension (42.2%), anemia (36.9%), hypothyroidism (36.9%), leucopenia (31.6%), and nausea (31.6%). Five (26.3%) patients experienced grade 3 TRAEs, including 1 case (5.3%) of anemia, 1 case (5.3%) of leukopenia, 1 case (5.3%) of hand-foot syndrome and 2 cases (10.5%) of intestinal bleeding. One patient underwent colostomy due to lower gastrointestinal bleeding. There were no grade 4 or 5 adverse events. A complete list of TRAE is shown in **Table 3**. There was 1 instance of dose delay of cadonilimab due to the patient experiencing Grade 2 immune myocarditis, and there was 1 instance of anlotinib dose reduction due to Grade 3 hand-foot syndrome. One patient discontinued bevacizumab after experiencing Grade 3 intestinal bleeding.

**Table 3:** Adverse events of treatment.

Adverse event	Total, No. (%)	No. (%)		
		Grade 1	Grade 2	Grade 3
Anemia	7 (36.9)	2 (10.5)	4 (21.1)	1 (5.3)
Leucopenia	6 (31.6)	5 (26.3)	0 (0)	1 (5.3)
Thrombocytopenia	3 (15.8)	3 (15.8)	0 (0)	0 (0)
ALT increased	2 (10.5)	2 (10.5)	0 (0)	0 (0)
AST increased	4 (21.1)	4 (21.1)	0 (0)	0 (0)
Urinary occult blood Positive	4 (21.1)	4 (21.1)	0 (0)	0 (0)
Proteinuria	9 (47.4)	6 (31.6)	3 (15.8)	0 (0)
Creatinine increased	1 (5.3)	0 (0)	1 (5.3)	0 (0)
Bilirubin increased	5 (26.3)	5 (26.3)	0 (0)	0 (0)
Hypothyroidism	7 (36.9)	7 (36.9)	0 (0)	0 (0)
Hyperthyroidism	1 (5.3)	1 (5.3)	0 (0)	0 (0)
Nausea	6 (31.6)	4 (21.1)	2 (10.5)	0 (0)
Fatigue	5 (26.3)	5 (26.3)	0 (0)	0 (0)
Rash	1 (5.3)	1 (5.3)	0 (0)	0 (0)
Hand-foot syndrome	2 (10.5)	0 (0)	1 (5.3)	1 (5.3)
Pyrexia	1 (5.3)	1 (5.3)	0 (0)	0 (0)
Intestinal bleeding	2 (10.5)	0 (0)	0 (0)	2 (10.5)
abdominal pain	1 (5.3)	0 (0)	1 (5.3)	0 (0)
Hyperglycemia	4 (21.1)	4 (21.1)	0 (0)	0 (0)
Hypertension	8 (42.2)	4 (21.1)	4 (21.1)	0 (0)
Immune myocarditis	1 (5.3)	0 (0)	1 (5.3)	0 (0)
Immune myositis	1 (5.3)	1 (5.3)	0 (0)	0 (0)

## Discussion

The management of r/mCC has been a challenge due to the limited variety of available treatment options. The discovery of novel treatments that prolong survival without inducing toxicity is crucial for improving the clinical management and outcomes in patients with r/mCC. Results from our study demonstrate that a combined immunologic and antiangiogenic regimen has both promising efficacy and acceptable safety with an ORR of 21.1% and DCR of 42.1%. The median PFS was 10.5 months.

In the GOG 240 trial, bevacizumab, in combination with chemotherapy, demonstrated a survival benefit and is now proven as an effective first-line therapy in the treatment of r/mCC (6). The combined regimen showed a median PFS of 8.2 months and a median OS of 17 months. However, the maintenance of this combined treatment was poor. In our study, the median PFS of cadonilimab plus anti-angiogenesis in heavily pretreated r/mCC was 10.5 months and the median OS has not yet been reached. These results show promising efficacy and provide a new treatment option for r/mCC patients. Notably, more than half of our study’s patients (57.9%) had previously received at least 2 lines of systemic therapy which suggests that this drug combination holds promise for heavily pretreated r/mCC.

Since immune escape by tumor cells through the expression of PD-L1, immunotherapy targeting PD-L1 expression has shown promising efficacy in advanced cervical cancer (19). Additionally, PD-L1 levels have been shown to predict the efficacy of immunotherapy (19). Similarly, our results showed an improved efficacy trend for patients with high levels of PD-L1 expression, with an DCR 50% in PD-L1 positive patients and 25% in PD-L1 negative patients. Due to the limited number of patients with assessed PD-L1 status, data analysis was not possible.

Based on the KEYNOTE-028 and KEYNOTE-158 studies, the U.S. Food and Drug Administration (FDA) approved pembrolizumab, a PD-1 inhibitor, for the second-line treatment of r/mCC in 2018. However, in phase II of the KEYNOTE-158 clinical trial, the ORR of pembrolizumab in patients with r/mCC was only 12.2% and the DCR was 30.6% (20), which was much lower than our study’s findings (ORR of 21.1% and DCR of 42.1%). Of note, more than half of our patients (63.2%) had failed previous treatment of PD-1 inhibitors. Collectively, these findings imply that combined anti-PD-1 and CTLA-4 inhibition enhances antitumor activity and the use of cadonilimab combination therapy might remain potentially effective despite the previous failure of PD-1 inhibitors. However, we should emphasize that due to the limited sample size, these findings are very preliminary and should be interpreted with caution.

Based on the first interim analysis of the phase III KEYNOTE-826 study (21), pembrolizumab combined with platinum-based chemotherapy ± bevacizumab was approved as first-line treatment for patients with PD-L1- positive persistent cervical cancer or r/mCC (CPS ≥ 1). Median PFS was significantly longer in the pembrolizumab arms in patients with PD-L1-positive tumors with CPS ≥ 1 (median 10.4 vs 8.2 months), which was slightly shorter than that of our study (median PFS of 10.5 months). Due to the limitation of sample size in this study, it is difficult to conclude that our research was superior to the KEYNOTE-826 study. However, the longer PFS suggests that this approach was optional in patients with heavily pretreated r/mCC.

Cadonilimab (AK104) is the first dual immune checkpoint inhibitor to be approved for use in many types of cancers (14, 15, 22). Results of a multicenter, open-label, phase 1b/2 study of cadonilimab monotherapy in patients with previously treated r/mCC from Gao et al. showed that the ORR was 32.3% in all patients and 42.9% in PD-L1-positive patients(15). The ORR was higher than a phase II trial study of balstilimab (anti–PD-1) combined with zalifrelimab (anti–CTLA-4) as second-line treatment for patients with r/mCC (ORR of 25.6%) (23), and it was also higher than PD-1 inhibitor monotherapy (24). Due to the aforementioned results, cadonilimab was approved in China as monotherapy for the treatment of patients with r/mCC after failed platinum-based chemotherapy in June 2022. The fact that our study’s ORR was lower than Gao et al’s was likely due to the following reasons: firstly, patients in our study were heavily pretreated, with 57.9% of our patients previously receiving at least 2 lines of systemic therapy and 47.4% receiving prior treatment with an anti-PD-1 inhibitor. Patients who had previously received immunotherapy were excluded from Gao’s study. Secondly, the treatment regimen in our study was given irrespective of PD-L1 expression status. Lastly, the observed difference in ORR may be due this study’s limited sample size. However, the combination of cadonilimab and anti-angiogenic therapy holds promise, with an ORR of 21.1% and DCR of 42.1% for heavily pretreated r/mCC. Thus, we believe that our research might provide a potentially effective novel treatment option for r/mCC, regardless of PD-L1 status.

Combination treatment in our study was well tolerated. No patients underwent discontinuation of cadonilimab therapy. Two patients (10.5%) discontinued bevacizumab due to Grade 3 intestinal bleeding. The overall rate of any-grade TRAEs (81.3%) was comparable with the any-grade TRAE rate reported for sintilimab plus anlotinib in r/mCC patients (18), and it was lower than the rate from a recent study of cadonilimab combined with standard therapy of r/mCC (95.6%) (16). The most common TRAEs were proteinuria, hypertension, anemia, hypothyroidism, leukopenia, and nausea. There were no grade 4 or 5 adverse events. Grade 3 TRAEs were noted in 26.3% of patients, which was much lower than the rates seen in the CLAP trial (54.5%), a phase 3 trial (66.9%) of lenvatinib plus pembrolizumab for advanced endometrial cancer, and a recent phase II study (51.0%) on the treatment of r/mCC with cadonilimab combined with standard therapy (16, 25). These findings suggest that cadonilimab, in combination with bevacizumab or anlotinib, was safe and well tolerated. No fistula occurred in our study. It should be noted that the use of bevacizumab would increase the risk of fistula in patients who had been previously irradiated. Experience from our institution suggested that patients who have received prior radiotherapy and have local recurrence shortly (< 6 months) should be carefully evaluated for treatment benefit and risk of fistula occurrences before using antiangiogenic therapy, especially bevacizumab. Considering that all patients in our study had received prior radiotherapy, bevacizumab was administered with a reduced dose of 7.5 to 10 mg/kg intravenously according to the assessment of the patient’s risk of fistula and patient’s economic factors.

To our knowledge, this is the first study to evaluate the combination of cadonilimab and antiangiogenic therapy in heavily pretreated patients with r/mCC patients. The finding suggests this regimen could be potentially efficacious and safe with relatively manageable toxicity. But we need emphasize that our findings are very preliminary and exploratory with several limitations, such as the small sample size, the self-limitation of the retrospective study design, and insufficient follow-up time. Despite these limitations, we believe that our findings are valuable, as they might provide a potentially effective treatment for heavily pretreated r/mCC. Further prospective clinical trials are needed to confirm our findings.

At the same time, we also recommend exploring combination schedules to improve overall efficacy, such as adjusting the timing of administration of cadonilimab and bevacizumab or exploring their combination with other targeted drugs.

## Conclusion

In conclusion, the combination of cadonilimab plus anti-angiogenesis therapy might have potential benefits in patients with r/mCC who have failed at least 1 previous line of treatment, including previous failure of PD-1 inhibitors, with tolerable side effects. Nevertheless, future larger, well-designed, and prospective studies are needed for validation of our findings.

## Data Availability

The raw data supporting the conclusions of this article will be made available by the authors, without undue reservation.
